# Computational study of novel natural inhibitors targeting aminopeptidase N(CD13)

**DOI:** 10.18632/aging.103155

**Published:** 2020-05-09

**Authors:** Junliang Ge, Zhongfeng Wang, Ye Cheng, Junan Ren, Bo Wu, Weihang Li, Xinhui Wang, Xing Shu, Ziling Liu

**Affiliations:** 1 Clinical College, Jilin University, Changchun, China; 2 Hepatopancreatobiliary Medicine Department, Jilin University First Hospital, Changchun, China; 3 Department of Neurosurgery, The Xuanwu Hospital Capital Medical University, Changchun, Beijing, China; 4 Department of Orthopedics, The First Hospital of Jilin University, Changchun, China; 5 Department of Orthopaedic Surgery, Xijing Hospital, The Fourth Military Medical University, Xi'an, China; 6 Department of Oncology, the First Hospital of Jilin University, Changchun, China; 7 The Laboratory of Cancer Precision Medicine, The First Hospital of Jilin University, Changchun, China; 8 Department of Oncology, The First Hospital of Jilin University, Changchun, China

**Keywords:** cancer, discovery studio, aminopeptidase N(CD13), bestatin

## Abstract

Objectives: To screen and identify ideal leading compounds from a drug library (ZINC15 database) with potential inhibition of aminopeptidase N(CD13) to contribute to medication design and development.

Results: Two novel natural compounds, ZINC000000895551 and ZINC000014820583, from the ZINC15 database were found to have a higher binding affinity and more favorable interaction energy binding with CD13 with less rodent carcinogenicity, Ames mutagenicity, and non-inhibition with cytochrome P-450 2D6. Molecular dynamics simulation analysis suggested that the 2 complexes, ZINC000000895551-CD13 and ZINC000014820583-CD13, have favorable potential energy, and exist stably in the natural circumstances.

Conclusion: This study discovered that ZINC000000895551 and ZINC000014820583 were ideal leading compounds to be inhibitions targeting to CD13. These compounds were selected as safe drug candidates as CD13 target medication design and improvement.

Materials and Method: Potential inhibitors of CD13 were identified using a series of computer-aided structural and chemical virtual screening techniques. Structure-based virtual screening was carried out to calculate LibDock scores, followed by analyzing their absorption, distribution, metabolism, and excretion and toxicity predictions. Molecule docking was employed to reveal binding affinity between the selected compounds and CD13. Molecular dynamics simulation was applied to evaluate stability of the ligand-CD13 complex under natural environment.

## INTRODUCTION

Cancer is a lethal condition ranking the second among all factors that cause human death in the world. The treatment has evolved to include different modalities including surgery, chemotherapy, radiotherapy and immunotherapy [1, 2]. Despite these different treatment approaches, cancer has distinctive common characters including metastasis, adhesion and vascular hyperplasia. There is still a lack of a broad-spectrum approach to cancer treatment targeting these characters.

CD13, also known as the aminopeptidase N(APN), belongs to the M1 metalloprotease family, and it is common and widespread. CD13 can residue from the peptide hormone angiotensin III (AngIII) to generate angiotensin IV (Ang IV), which has been shown to cause vasodilatation, hypertrophy, and activation of NF-κB. [3–5] CD13 plays important roles in vascular endothelial morphogenesis during angiogenesis, through increasing the cellular adhesion to various adhesion molecules including type IV collagen, type I collagen and fibronectin [6]. CD13 could be up-regulated by hypoxia, angiogenic factors such as vascular endothelial cell growth factor (VEGF), basic fibroblast growth factor (bFGF), and type IV collagen facilitates the migration of these cells into the surrounding tissue [7]. Moreover, the high levels of CD13 expressed on the surface of the cells may oppose the apoptosis of the leukemic cells, leading to the overgrowth of the cells [8].

Over all, CD13 is related to the metastasis, adhesion, vascular hyperplasia and cell apoptosis, which are all related with tumorigenesis. Tumor angiogenesis and metastasis can be suppressed by inhibiting its activity. Many studies have revealed that CD13 is related with various kinds of cancers such as melanoma, colon cancer, prostate cancer, lung cancer, liver cancer, leukemia, thyroid cancer, etc. [9–14] Therefore, an efficient leading compound inhibiting CD13 can be exploited therapeutically. Recent years, some studies had reported some compounds that could be inhibitors to CD13. A kind of inhibitors of CD13, Bestatin, is the most mature research, and has already been marketed. Bestatin, also known as ubenimex, was reported synergistically enhanced the antitumor effects in liver cancer treatment via inhibiting CD13 [15, 16]. Therefore, CD13 inhibitors are potential adjuvants for antineoplastic therapies. The aim of this study was to find some other natural compound inhibitors from a natural drug library more effective than Bestatin in cancer treatment.

Nowadays, natural products and their derivatives play a decisive role in pharmacologic market, because of their unique molecular structures and potential biologic function, which have made a great contribution to the design and improvement of medicine [17, 18]. Several recent publications have reported the importance of CD13 in occurrence and development of cancer and its inhabitation’s effect in cancer combined therapy [19, 20]. This study conducted a series of structural biologic and chemistry methods (e.g., virtual screening, molecule docking, toxicity prediction) to screen and identify compounds that had potential inhibiting functions related to CD13. Additionally, our study predicted absorption, distribution, metabolism, excretion, and toxicity of selected significant compounds. This study provides lists of candidate compounds from the ZINC15 database and their pharmacologic properties, which could provide a solid foundation in developing CD13 inhibitor research. The significance of this study is to find Potential compounds as CD13 inhibitors to establish the foundation of medication development and compound improvement in cancer curing.

## RESULTS

### Virtual screening of natural products database against CD13

The S1 pocket is an important regulatory site of CD13, as the Val (P1) side chain in the Ang IV complex binding to this active site could cause vasodilatation, hypertrophy, and stimulate NF-κB pathway, and then neovascularization occurs. Therefore, this pocket region was selected as a reference site. A total of 17,931 biogenic-for sale-named product molecules were taken from the ZINC15 database. Chemical structure of the receptor protein, CD13, was selected to test the pharmacologic properties of other compounds. One inhibitor, Bestatin, was chosen as the reference compound. After screening, 4761 compounds were found to be eligible to bind stably with CD13; among those, 318 compounds were found to have higher LibDock scores than Bestatin. (LibDock score: 97.2164), which are listed in Supplementary Table 1. The top 20 ranked compounds are listed in Table 1.

**Table 1:** Top 20 ranked compounds with higher LibDock.

**Number**	**Compounds**	**LibDock score**
1	ZINC000006094124	127.999
2	ZINC000014820552	125.435
3	ZINC000006092198	125.32
4	ZINC000018185774	125.143
5	ZINC000000899884	125.018
6	ZINC000018185774	124.894
7	ZINC000014820405	123.912
8	ZINC000020470300	122.885
9	ZINC000005732375	122.783
10	ZINC000028465419	122.38
11	ZINC000005732375	122.275
12	ZINC000006092239	122.243
13	ZINC000003871576	121.97
14	ZINC000013481874	121.889
15	ZINC000003871576	121.855
16	ZINC000000155807	121.659
17	ZINC000014820583	121.366
18	ZINC000005732375	120.855
19	ZINC000000895551	120.767
20	ZINC000018169010	120.704

### ADME and toxicity prediction

Pharmacologic properties of all the 20 selected ligands with Bestatin were predicted using the ADME module of Discovery Studio 4.5, including aqueous solubility level, blood-brain barrier level, CYP2D6 binding, hepatotoxicity, human intestinal absorption level, and plasma protein binding properties (Table 2). The aqueous solubility prediction (defined in water at 25°C) indicated that all the compounds were soluble in water. Only one compound, ZINC000018185774, was predicted to be inhibitor with CYP2D6, which was an essential enzyme in drug metabolism. As to hepatotoxicity, all compounds were found to be toxic, while Bestatin is nontoxic. For human intestinal absorption, except 2 compounds, ZINC000006092198 and ZINC000018169010, other ligands had as absorption level as good as Bestatin did. Plasma protein binding properties indicated that ZINC000006094124, ZINC000014820552, ZINC000006092198, ZINC000000899884, ZINC000014820405, ZINC000020470300, ZINC000006092239, ZINC000014820583 and ZINC00000 5732375 had good absorption, while other 11 compounds had weak absorption.

**Table 2:** Adsorption, distribution, metabolism, and excretion properties of compounds.

**Number**	**Compounds**	**Solubility level**	**BBB level**	**CYP2D6**	**Hepatotoxicity**	**Absorption level**	**PPB level**
1	ZINC000006094124	3	3	0	1	0	1
2	ZINC000014820552	2	2	0	1	0	1
3	ZINC000006092198	3	4	0	1	1	1
4	ZINC000018185774	4	3	0	1	0	0
5	ZINC000000899884	2	2	0	1	0	1
6	ZINC000018185774	3	4	1	1	0	0
7	ZINC000014820405	3	3	0	1	0	1
8	ZINC000020470300	2	1	0	1	0	1
9	ZINC000005732375	3	3	0	1	0	0
10	ZINC000028465419	2	2	0	1	0	0
11	ZINC000005732375	3	3	0	1	0	0
12	ZINC000006092239	3	3	0	1	0	1
13	ZINC000003871576	4	3	0	1	0	0
14	ZINC000013481874	4	4	0	1	0	0
15	ZINC000003871576	4	3	0	1	0	0
16	ZINC000000155807	3	4	0	1	0	0
17	ZINC000014820583	2	2	0	1	0	1
18	ZINC000005732375	3	3	0	1	0	1
19	ZINC000000895551	3	3	0	1	0	0
20	ZINC000018169010	4	4	0	1	3	0
21	ZINC000001532730	4	4	0	0	0	0

BBB, blood-brain barrier; CYP2D6, cytochrome P-450 2D6; PPB, plasma protein binding; O6-BG, O6-benzylguanine.

Aqueous-solubility level: 0, extremely low; 1, very low, but possible; 2, low; 3, good.

BBB level: 0, very high penetrant; 1, high; 2, medium; 3, low; 4, undefined.

CYP2D6 level: 0, noninhibitor; 1, inhibitor.

Hepatotoxicity: 0, nontoxic; 1, toxic.

Human-intestinal absorption level: 0, good; 1, moderate; 2, poor; 3, very poor.

PPB: 0, absorbent weak; 1, absorbent strong.

**Figure 1:**
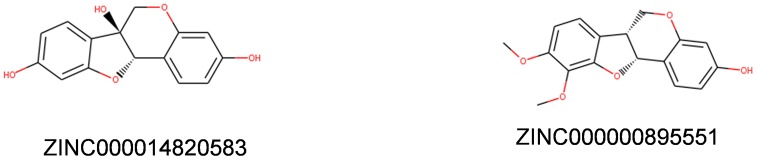
**Structures of novel compounds selected from virtual screening.**

Safety and toxicity should also need full consideration in this study. To confirm the safety of the top20 compounds, different kinds of toxicity indicators of these compounds and Bestatin, including rodent carcinogenicity (based on the U.S. National Toxicology Program dataset), developmental toxicity potential properties, Ames mutagenicity were predicted by using a computational method in the TOPKAT module of Discovery Studio 4.5 (Table 3). Results indicated that 13 compounds were found to be nonmutagenic, and 2 compounds showed not to have developmental toxicity potential(DTP), while one compound, ZINC000000895551, had little DTP. The reference Bestatin was predicted to have low rodent carcinogenicity and low DTP. Considering all the results in Table 3, ZINC000000895551 and ZINC000014820583 were identified to be the ideal leading compounds with non-CYP2D6 inhibitors and less rodent carcinogenicity, together with Ames mutagenicity, and developmental toxicity potential compared with other compounds. Therefore, ZINC000000895551 and ZINC000014820583 were confirmed as safe drug candidates and were selected for the subsequent research (Figure 1).

**Table 3:** Toxicities of compounds.

**Number**	**Compounds**	**Mouse NTP**		**Rat NTP**		**Ames**	**DTP**
		**Female**	**Male**	**Female**	**Male**		
1	ZINC000006094124	0	0	1	0.068	0	0.881
2	ZINC000014820552	0	0	1	0.63	0	0.997
3	ZINC000006092198	0	1	0	0.998	1	0.931
4	ZINC000018185774	0.001	1	1	0.995	1	0.999
5	ZINC000000899884	0	0	1	0.027	0	0.949
6	ZINC000018185774	0.001	1	1	0.995	1	0.999
7	ZINC000014820405	0.001	0	1	0.823	0	0.8
8	ZINC000020470300	0.001	1	0.989	1	0.01	1
9	ZINC000005732375	0.923	1	0	1	0.026	1
10	ZINC000028465419	0.013	0.999	0	1	0	1
11	ZINC000005732375	0.923	1	0	1	0.026	1
12	ZINC000006092239	0	0.019	0	0	1	1
13	ZINC000003871576	0.002	1	0	0.996	0.994	0.999
14	ZINC000013481874	0	0	0	0	0	0
15	ZINC000003871576	0.002	1	0	0.996	0.994	0.999
16	ZINC000000155807	0	0	1	1	0	1
17	ZINC000014820583	0	0	1	0.674	0	0.009
18	ZINC000005732375	0.921	1	0	1	0.015	1
19	ZINC000000895551	0	0	1	0.012	0	0.427
20	ZINC000018169010	0	1	0	1	1	1
21	ZINC000001532730	0	0	0	0	0	0

NTP, U.S. National Toxicology Program; DTP, developmental toxicity potential.

NTP<0.3 (noncarcinogen); >0.8 (carcinogen).

Ames<0.3 (nonmutagen); >0.8 (mutagen).

DTP<0.3 (nontoxic); >0.8 (toxic).

### Analysis of ligand binding and ligand pharmacophore

**Figure 2:**
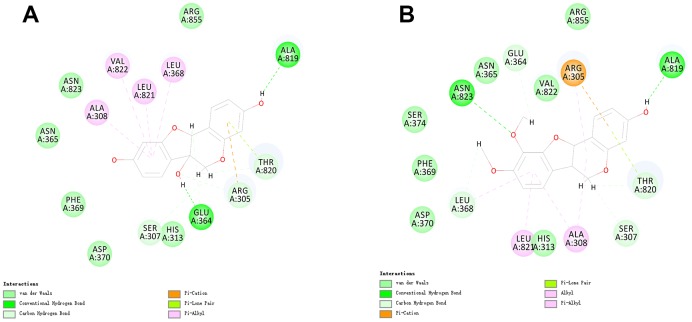
Schematic of intermolecular interaction of the predicted binding modes of (**A**) ZINC000014820583 with aminopeptidase N(CD13), (**B**) ZINC000000895551 with CD13.

**Figure 3:**
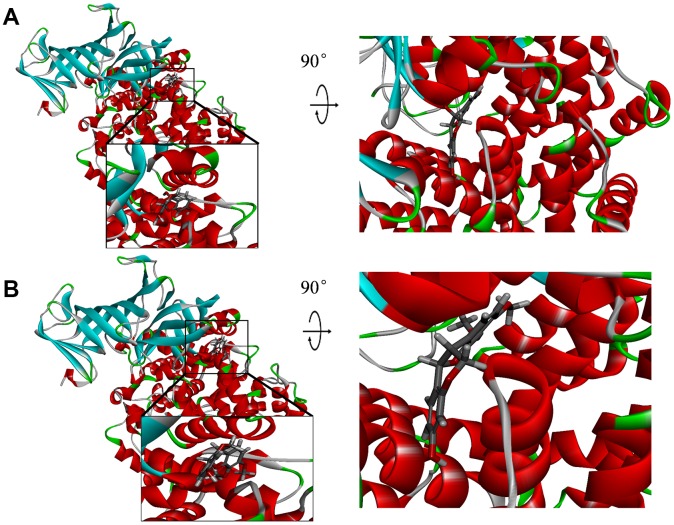
**Schematic drawing of interactions between ligands and aminopeptidase N(CD13).** Blue represents positive charge; red represents negative charge; and ligands are shown in sticks, with the structure around the ligand-receptor junction shown in thinner sticks. (**A**) ZINC000014820583-CD13 complex. (**B**) ZINC000000895551-CD13 complex.

To identify ligand binding mechanisms of these compounds and Bestatin, ZINC000000895551 and ZINC000014820583 were docked into the molecule structure of CD13 using CDOCKER module, and CDOCKER potential energy was calculated and displayed in Table 4. RMSD between the docked pose and the crystal structure of the Bestatin and CD13 complex was 0.75 Å, proving that the CDOCKER module applied in this study was strongly reliable for reproducing the experiment. As results shown in Table 5, the CDOCKER potential energy of ZINC000000895551 and ZINC000014820583 were significantly lower than the reference ligand Bestatin (ZINC000001532730) (24.7496 kcal/mol), which suggested that these 2 compounds had a much higher binding affinity with CD13 than Bestatin. Hydrogen bonds and π-related interactions were also performed using structural computation study (Figures 2 and 3). Results showed that ZINC000000895551 formed 6 pairs of hydrogen bonds with CD13, by the H^28^ of the compound with ALA819:O of CD13, H^32^ of the compound with GLU364:OE2 of CD13, the O^20^ of the compound with ARG305:HD1, the H^25^ of the compound with GLU364:OE1, the H^30^ of the compound with SER307:OG and the H^31^ of the compound with THR820:O. Besides, 4 π-related interactions were formed in the complex, including the π-Orbitals between ZINC000000895551 and the ALA308, LEU368, LEU821, and VAL822 of CD13. ZINC000014820583 formed 6 pairs of hydrogen bonds with CD13, by the H^34^ of the compound with ALA819:O of CD13, the O^9^ of the compound with ASN823:HN of CD13, the H^24^ of the compound with LEU368:O of CD13, the H^30^ of the compound with GLU364:O of CD13, the H^36^ of the compound with SER307:OG of CD13 and the H^37^ of the compound with THR820:O of CD13. It also formed 5 pairs π-related interactions in the complex, consist of 2 pairs between ALA308 of CD13 and the compound, ARG305 of CD13 with the compound, LEU368 of CD13 with the compound, and LEU821 of CD13 with the compound. As to the reference compound Bestatin, it formed 2 hydrogen bonds with CD13, by the H^38^ of the compound with THR820:OG1 of CD13, and H^36^ of the compound with THR820:O of CD13. Also three π-related interactions formed between Bestatin and CD13, including ALA308, LEU368 and LEU821 of CD13 with Bestatin.

**Table 4:** CDOCKER potential energy of compounds with CD13.

**Compound**	**CDOCKER potential energy (kcal/mol)**
ZINC000000895551	-44.2913
ZINC000014820583	-58.4829
ZINC000001532730	24.7496

**Table 5:** Hydrogen bond interaction parameters for each compound with CD13 residues.

**Receptor**	**Compound**	**Donor atom**	**Receptor atom**	**Distances (Å)**
CD13	ZINC000000895551	A:ALA819:O	ZINC000000895551:H28	1.99
		A:GLU364:OE2	ZINC000000895551:H32	2.56
		A:GLU364:OE1	ZINC000000895551:H25	2.44
		A:SER307:OG	ZINC000000895551:H30	3.00
		A:THR820:O	ZINC000000895551:H31	2.27
	ZINC000014820583	A:ALA819:O	ZINC000014820583:H34	2.01
		A:GLU364:O	ZINC000014820583:H30	2.47
		A:SER307:OG	ZINC000014820583:H36	2.92
		A:THR820:O	ZINC000014820583:H37	2.24
	ZINC000001532730	A:THR820:OG1	ZINC000001532730:H38	2.86
		A:THR820:O	ZINC000001532730:H36	2.31

### Molecular dynamics simulation

**Figure 4:**
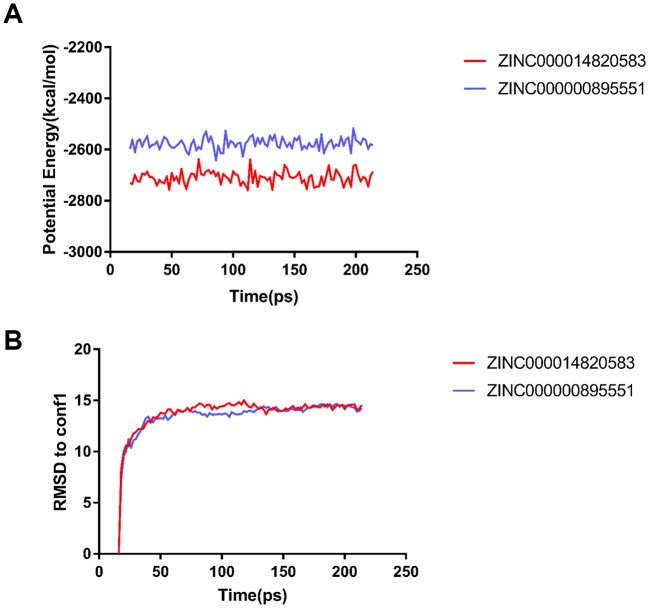
**Results of molecular dynamics simulation of the compounds ZINC000014820583 and ZINC000000895551.** (**A**) Potential energy. RMSD, root-mean-square deviation. (**B**) Average backbone root-mean-square deviation.

In order to estimate the stabilities of the ligand-CD13 complexes on natural environment conditions, the molecular dynamics simulation module test was conducted. According to the original conformations from the molecular docking experiment through CDOCKER module, RMSD curves and potential energy chart of each complex were shown in Figure 4. The trajectories of all complexes achieved equilibrium after 18 ps; RMSD and potential energy of these complexes tends to stability with time. The molecular dynamics simulation result suggested that these hydrogen bonds and π-related interactions formed by compounds and CD13 contributed to the stability of these complexes. Results showed that ZINC000000895551 and ZINC000014820583 could interact with CD13, and their complexes with CD13 could exist in a natural environment steadily, and act as regulatory roles to CD13 just like the reference Bestatin did.

## DISCUSSION

Cancer is the second most frequent cause of death worldwide in spite of significant improvements in cancer therapies during past decades. Research on the prevention and treatment of cancer has attracted worldwide attention [25]. Past researches has proved that vascular hyperplasia and endothelial damage of tumor are the major reason of cancerization and metastasis of tumors, and the main ways for cancers to get nutrition [26]. CD13, also named APN, which was found existing on many types of tissue in human body, contributes to several pathophysiological changes such as vascular hyperplasia and verification response through activating Ang IV and involving in pathways including NF-κB, bFGF and VEGF [27, 28]. Therefore, to find an inhibitor of CD13 to weaken its activity is a key point to resist the development and metastasis of cancer. In recent years, the development of CD13 inhibitors used in clinical applications and its combination with different anticancer drugs aiming to improve the effect of chemotherapy had become a research hotspot in the field of cancer chemotherapy [29, 30]. Although great discoveries with compounds have been made as CD13 inhibitor drug designing and development, only 1 inhibitor, Bestatin, the ligand selected as the reference drug in this study, has been part of relatively mature research until now [16, 31, 32]. However, there are still some limitations for Bestatin.

Though CD13 inhibitors have therapeutic possibility in anticancer field, there are significant limitations of Bestatin, currently being tested as potential anticancer compound. Relevant research showed that concentration level of serum Bestatin depends on its administration; when administered via the i.p., s.c., i.m., or p.o. routes, Bestatin had short serum t_1/2_, no more than 20 min; while administered by i.v. administration, Bestatin has a high initial serum level [33]. This study result suggest that Bestatin, as a chemotherapeutic substrate or concomitant chemotherapeutic drug, is hard controlling its serum content, which means more frequent administration and more attention to blood drug level monitoring. This fact is bound to bring difficulties to clinical anticancer application. Consequently, a kind of new CD13 inhibitor is urgently need in anticancer therapy to improve the effect of chemotherapy.

In this study, 17,931 biogenic-for sale-named product molecules taken from the ZINC15 database for virtual screening, was followed by ADME, TOPKAT, CDOCKER, and molecular dynamics simulation. LibDock scores unfolded degree of energy optimization and stability of the conformation. High LibDock score compounds illustrated better energy optimization and a stable conformation than lower score achievers. After calculation by the LibDock module, 4761 compounds showed to be capable to bind stably with CD13. Besides, among these ligands, 318 compounds had higher LibDock scores than Bestatin (LibDock score: 102.86), indicating that these 318 compounds could form a more stable complex with CD13 with better energy optimization compared with Bestatin. Based on LibDock score, the top 20 natural compounds were selected and pooled for further study.

ADME and toxicity predictions of the selected compounds were carried out to assess the pharmacologic properties of these compounds. The result suggested that ZINC000000895551 and ZINC000014820583 were confirmed as ideal candidacy compounds because of their good solubility in water and good intestinal absorption level. Furthermore, ZINC000000895551 and ZINC000014820583 were predicted to be no inhibitors of CYP2D6, no Ames mutagenicity, and less rodent carcinogenicity and developmental toxicity potential by comparison with other compounds. This information illustrates that these 2 compounds have preferable safety, and they are enough to be considered as potential ideal leading compounds. All in all, ZINC000000895551 and ZINC000014820583 were reasonably identified as relatively high quality drug candidacies. Results data above elucidated their potential use in drug designing. In spite of the toxicities or negative effects of the remaining drugs on the list, they also had prospective application in drug development by adding or deleting particular functional groups and atoms to reduce toxicity. Combining the above results, ZINC000000895551 and ZINC000014820583 were identified as ideal leading compounds, followed by further analysis was performed.

Following study revealed Ligand binding mechanism and chemical bonds of candidate compounds with CD13. CDOCKER module computation confirmed that CDOCKER interaction energy of ZINC000000895551 and ZINC000014820583 was obviously lower than the reference ligand Bestatin, which demonstrates why these 2 compounds may have a higher binding affinity with CD13 compared with Bestatin. Then, the chemical structures of the 2 compounds were analyzed by molecular structure inspection. The 2 complexes that CD13 combined with 2 candidacies have more chemical bonds than Bestatin, which again indicates that these 2 compounds could bind with CD13 at active site S1 pocket more stably. So they may contribute to competitive inhibition of activity of CD13 and act in improved the effect of cancer treatment.

Finally, their stabilities in the natural environment were assessed performing molecular dynamics simulation. Computational results of RMSD and potential energy of these ligand-CD13 complexes showed that the trajectories of complexes reached equilibrium after 18 ps. RMSD and potential energy of these complexes tends to stability with time, which suggested that these 2 complexes could exist in the natural environment stably. Based on these results, drug designation and development, such as modification and refinement, could be prospectively carried out to make combination of ligand and receptor more stable. Moreover, it is also noteworthy that agonist and inhibitor always share a similar chemical skeleton structure, and adding or deleting different groups or atoms leads the opposite effects. As a result of their significantly pharmacologic properties, highly binding affinity and stabilization combining with CD13, natural compounds identified in this research could provide a valuable resource for CD13-related drug development.

In recent years, design and development of oncology drugs are hot research topics worldwide. However, overall progress has not seemed encouraging. This study elucidated that the first and the most important step in drug designation was to identify ideal leading compounds. In this research, 5 modules of Discovery Studio 4.5, including LibDock, ADME, TOPKAT, CDOCKER, and molecular dynamics simulation, were applied to screen and analyze the biochemical structure properties of novel potential compounds. Molecule conformation, pharmacologic properties, binding affinity, and stability of each selected compound were also fully tested and calculated to determine their advantages compared with the reference compound, Bestatin. A series of high-tech computational studies showed that these 2 compounds may have potential effect in drug therapy of multiple types of cancer. However, it is noteworthy that no single natural compound could be directly marketed without thousands of refinement and improvement. On the basis of the identification of these 2 leading compounds with the most potential, further research could directly concentrate on improvement and refinement of them. Furthermore, our study provides a guideline for screening leading compounds with potential therapy effect. Through this high-tech method, more leading compounds could be screened, which could improve current drug development and bring increase of efficiency.

Although this study was conducted by elaborate design, it still has some limitations. Further experiments, such as animal testing, need to be performed to verify our results more firmly, and more indicators, such as half-maximal inhibitory concentration and half-maximal effective concentration, should be assessed in future studies to test more compounds properties.

## CONCLUSIONS

This study performed a series of computer-aided structural and chemical analysis technology (including virtual screening, molecule docking, ADME, toxicity prediction) to screen and identify the ideal leading compounds with functions to inhibit CD13. Two compounds, ZINC000000895551 and ZINC000014820583, were selected as safe candidacies, and they had great significance in CD13 inhibitor medication development. Furthermore, this study provided a list of drug candidates and their pharmacologic properties, which may contribute to CD13 or other proteins in medication design and improvement.

## MATERIALS AND METHODS

### Discovery studio software and ligand library

Discovery Studio 4.5 software (BIOVIA, San Diego, California, USA) is a suite of software for simulating small molecule and macromolecule systems; it is a new generation of molecular modeling and environmental simulation software for the life sciences field, which aims to provide protein modeling, optimization, and drug design tools by applying protein structure and structural biologic computation. Numerous leading compounds and drug candidates were identified and refined through this method. LibDock module of Discovery Studio was employed for virtual screening; CDOCKER module was used for docking study; and ADME module was analyzed for pharmacologic properties. The Natural Products database in the ZINC15 database was selected to screen STING agonists. The ZINC15 database is a free database of commercially available compounds provided by the Irwin and Shoichet Laboratories, Department of Pharmaceutical Chemistry, University of California, San Francisco (San Francisco, California, USA).

### Structure-based virtual screening using LibDock

**Figure 5:**
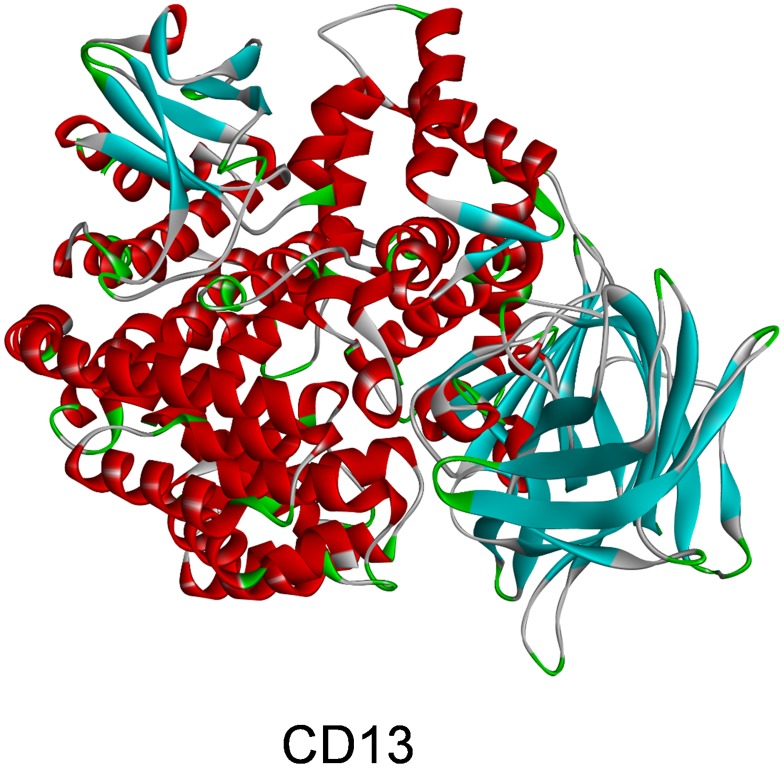
**Molecular structure of aminopeptidase N(CD13).**

Ligand-binding pocket region of CD13 was selected as the binding site to screen potential compounds to inhibit CD13. Virtual screening was carried out using the LibDock module of Discovery Studio 4.5 [21]. LibDock is a rigid-based docking module. It calculates hotspots for the protein through a grid placed into the binding site and polar and apolar probes. The hotspots are further used to align the ligands to form favorable interactions. The Smart Minimiser algorithm and CHARMM force field (Harvard University, Cambridge, Massachusetts, USA) were performed for ligand minimization, and all ligand poses were ranked based on the ligands score. The 1.9-Å crystal structure of human CD13 (Protein Data Bank identifier: 4fyq) was downloaded from the Protein Data Bank and the inhibitor Bestatin (Zinc15 database identifier: zinc000001532730) was downloaded from Zinc15 database, and they were imported to the working circumstance of LibDock. The chemical structure of CD13 is shown in Figure 5. The protein was prepared by serval steps including removing crystal water and other heteroatoms around it, followed by addition of hydrogen, protonation, ionization, and energy minimization. The CHARMM force field and the Smart Minimiser algorithm were applied for energy minimization [22]. The minimization performed 2000 steps with a root mean square gradient tolerance of 12.277, and the final root mean square gradient was 0.690. The prepared protein was prepared to define the binding site. Using the ligands (Bestatin) binding position, the active site for docking was generated. Virtual screening was performed by docking all the prepared ligands at the defined active site using LibDock. Based on the LibDock score, all the docked poses were ranked and grouped, and all compounds were ranked according to the LibDock score.

### Absorption, distribution, metabolism, and excretion and toxicity prediction

The ADME module of Discovery Studio 4.5 was employed to calculate absorption, distribution, metabolism, and excretion (ADME) of selected compounds, including their aqueous solubility, blood-brain barrier penetration, cytochrome P-450 2D6 (CYP2D6) inhibition, hepatotoxicity, human intestinal absorption and plasma protein binding level. TOPKAT module of Discovery Studio 4.5 was employed to calculate the toxicity and other properties of all the potential compounds, such as U.S. National Toxicology Program rodent carcinogenicity, Ames mutagenicity, developmental toxicity potential, and rat oral median lethal dose (LD50) and chronic oral lowest observed adverse effect level (LOAEL). These pharmacologic properties were fully considered in screening proper probable drug candidates for CD13.

### Molecule docking and pharmacophore prediction

CDOCKER module of Discovery Studio 4.5 was used for molecular docking study. CDOCKER is a molecular docking method based on CHARMM force field, which can produce high-precision docking results. The CHARMM force field was used for both receptors and ligands. Receptor is held rigid, while ligands are allowed to flex during the docking process. For each complex pose, the CHARMM energy and the interaction energy, which indicated ligand binding affinity, were calculated. Crystal structure of CD13 was obtained from the protein data bank. The crystal water molecules were generally removed in a rigid and semi-flexible docking process, causing the fixed water molecules to possibly affect the conformation of the receptor-ligand complex [23, 24]. The water molecules were removed, and hydrogen atoms were added to the protein. To prove the reliability of the analysis result, the initial inhibitor compound Bestatin was extracted from the Zinc15 database, the same to the Natural Products screened, and then Bestatin was docked into the crystal structure of CD13 to compare the root-mean-square deviation (RMSD) with these 2 conformations. The binding site sphere of CD13 was defined as the regions coming within 5-Å radius from the geometric centroid of the ligand Bestatin. During the docking process, the ligands were allowed to bind with the residues of protein groups within the binding site sphere. The structures of identified hits were prepared and docked into the binding site of CD13. Different poses of each ligand-CD13 complex were generated and analyzed on the basis of CDOCKER interaction energy.

### Molecular dynamics simulation

The best binding conformations of the ligand-CD13 complexes among the poses predicted by the molecule docking program were selected and prepared for molecular dynamics simulation. The ligand-receptor complex was put into an orthorhombic box and solvated with an explicit periodic boundary solvation water model. To simulate the physiologic environment, sodium chloride was added to the system with the ionic strength of 0.145. Then the system was subjected to the CHARMM force field and relaxed by energy minimization (500 steps of steepest descent and 500 steps of conjugated gradient), with the final root mean square gradient of 0.227. The system was slowly driven from an initial temperature of 296 K to the target temperature of 302 K for 2 ps, and equilibration simulations were run for 5 ps. Molecular dynamics simulation (production module) was performed for 25 ps with time step of 1 fs. The simulation was performed with the normal pressure and temperature system at a constant temperature of nearly 300 K during the process. The particle mesh Ewald algorithm was used to calculate long-range electrostatics, and the linear constraint solver algorithm was adapted to fix all bonds involving hydrogen. With initial complex setting as a reference, a trajectory was determined for RMSD, potential energy, and structural characteristics through the Discovery Studio 4.5 analysis trajectory protocol.

## Supplementary Material

Supplementary Table 1
